# Measuring the macroeconomic determinants of agricultural price volatility: Implications for natural resource commodity prices for green recovery

**DOI:** 10.3389/fpubh.2022.1035432

**Published:** 2022-12-16

**Authors:** Hang Liu

**Affiliations:** School of Management, Heilongjiang University of Science and Technology, Harbin, China

**Keywords:** natural resources prices volatility, FDI, inflation, human capital index, gross domestic product

## Abstract

With rapid growth, green economic recovery has been a key agenda for the globe. However, the price volatility for natural resources plays a significant role in reshaping the green recovery. Therefore, the current study investigates the impact of green recovery, hum, a capital index, GDP growth, foreign direct investment and inflation on natural resource volatility in China from 1995 to 2020. In order to investigate the long-term association among selected variables, this study employs the Autoregressive Distributive Lag (ARDL) model. In addition, the current research uses the Aikaik information (AIC) criteria for the model selections. Obtained outcomes show the significant contribution of green recovery, human capital, GDP growth, FDI and inflation increase the natural resource price volatility level. However, to validate the results of ARDL, this study also used the ECM approach and validated the prior findings. On behalf of outcomes, the current study implies some imperative policies to attain the desired objective for green growth.

## Introduction

In the present context, SDGs are a vital policy for all governments. Ecological economists have suggested the concept of “green recovery” to address climate change (1, 2). Important principles for post-COVID-19 sustainability-related transformations may be found in the bio-, circular-, and green economies, and the concept of “green growth” is becoming an increasingly central concern among academics (3–5). Policies implemented to combat the socioeconomic crises related to the pandemic and climate change is commonly referred to as “green recovery,” a phrase used to emphasize the strategic importance of sustainability in developing the future society (6). Businesses are being pushed to adapt to the changing needs of their customers, many of whom are concerned about the environment's state. The advent of green innovation can bring new prospects with a significant emphasis on environmental issues (7).

However, the natural resources are performing well to boost growth. Therefore, natural and mineral resources are considered a boon to a country's economy (8). The academic literature debunks the widespread belief that a country rich in natural resources will experience rapid economic development (9, 10). For decades, people have questioned and investigated whether or not natural resources are a boon or a bane (11). However, there are two different concepts of natural resources “natural resource abundance” and “resource curse” (12). The idea of a “resource curse” is predicated on the observation that countries rich in natural resources grow more slowly than those with fewer. Economists have found that having an excess of natural resources negatively influences the economy, although there are notable outliers like Australia and the USA (13).

By capitalizing on their comparative advantages, industrialized nations have been able to boost their economies, raise their per capita incomes, and boost other development indices throughout the history of the global economy (14).

Oil, minerals, and agricultural products are just some of the natural resources that can profoundly impact a country's economy and are therefore regarded as a gift from the earth (15). Literature has cast doubt on the assumption that a nation with a high concentration of natural resources will have faster economic expansion (16–19). In recent decades, researchers and thinkers have pondered whether or not natural resources are a boon or a bane (14). Ones that are well-endowed with natural resources are observed to have lower economic growth rates than countries that lack such abundance (20). According to the study's authors, there is a negative relationship between the availability of natural resources and economic growth (21). It's easy to think of countries like the United States and Australia as outliers (22). Western European and Asian economies, for example, have higher growth rates than resource-rich countries like Brazil and Venezuela (23). New 21st-century breakthroughs in technology, finance, and the economy raise questions about the viability of the “slow growth” hypothesis and the countries particularly rich in natural resources.

Other considerations, such as spending on human capital, may also drive sustainable resource models. An organization's ability to compete, maintain market share, and create value relies on its people, resources and intellectual capital (24), which results from their reports that HR is a key driver of business results. Manzoor et al. (18) argued that companies could benefit from investing in human capital by gaining access to new areas of expertise. The education of human capital to understand the value of conservation is one possible route. While human capital's importance to business success is widely acknowledged, its potential to curb carbon emissions at the firm level is often overlooked. Human capital was crucial in lowering carbon emissions after a large-scale analysis of the correlation between human resources and environmental deterioration (11).

Private capital inflows in the form of FDI have also increased throughout this time, particularly from emerging economies like China (25). Overall gains in employment and welfare from foreign capital inflows have been limited, as has been the case in other developing regions. This is largely attributable to the fact that these inflows are mostly directed toward capital-intensive natural resource exploitation. The connections between foreign direct investment and natural resources, and to a lesser extent, natural resources and capital flight, have received much study in the economics literature. However, the potential impact of natural resources in connecting capital flight and FDI has received scant study. Still, natural resources may provide conceptual and empirical explanations for why FDI and capital flight often move together. Most foreign direct investment (FDI) in African nations goes toward extractive industries, which have minimal ties to the home economy, are capital-demanding, and produce few jobs. This may explain why FDI has low spillover effects on the domestic economy. Additionally, natural resources can facilitate the illegal entrance of foreign cash motivated by rent-seeking activities (26).

It is necessary to highlight the contribution of this study to existing literature, having a long debate about natural resources, human capital, green recovery, and foreign direct investment. Firstly, is there a relationship between FDI in selected economies and natural resource price volatility? On the one hand, it may be hypothesized that FDI may provide environmentally friendly resources, which would imply a positive relationship between the two phenomena. This question is worth investigating, given that natural resource price volatility has increased faster than other factors as a source of foreign resource inflows. Conversely, natural resource price volatility is linked to high levels of foreign direct investment (FDI), indicating an environment favorable to investment in the destination country. Secondly, human capital is considered a significant factor that could control price fluctuation concerning natural resources; therefore, this study tries to answer whether human capital can control natural resource price volatility. Thirdly, this study investigates the role of economic growth and inflation on natural resource price volatility. Fourthly, in order to investigate the proposed objectives, this study uses the Autoregressive Distributive Lag model (ARDL) and Fully Modified Ordinary Least Square (FMOLS). However, the current study proposes some policy implications for sustaining natural resources. It adds to the existing literature in a way that may encourage policymakers and future academics to take note. Being an important economic determinant that may affect the environment, this study fills a gap in the literature by presenting empirical evidence about the stated variable. Therefore, this article will pave the way for future scholars to investigate this relationship in established and emerging economies.

The remainder of the research is structured as follows: In Section Literature review, we present a comprehensive literature review on the relationship between natural resource price volatility and economic performance; in Section Data and methods, we define the data and variables of interest and the methodology for conducting empirical estimations; in Section Results and discussion, we present empirical results and discuss them; and in Section Conclusion and policy implications, we draw conclusions and discuss the study's policy implications.

## Literature review

In this section, the current study summarizes the existing studies concerning natural resources and other macro factors. However, this section is divided into four sub-sections.

### Green growth and natural resources price volatility

The commodity price index is the primary indicator of macroeconomic performance, with GDP serving as the corresponding element of the economy. The depreciation of fixed capital assets is a common source of GDP growth (27). Market-seeking motivations and natural resources in China were studied by Andersson and Börjesson (28). The importance of GDP is due to a number of interrelated elements, including natural resources and economic incentives. The influence of GDP has been analyzed using system-GMM, fixed effect, and random effect approaches. Researchers found that GDP has a stimulating and beneficial effect on the commodities index and volatility of natural resources. These results are at odds with those of studies by Umar et al. (29) and Bohl and Sulewski (30), who found that rising GDP led to greater productivity of natural resources on par with other commodities and a consequent decrease in natural resource prices. Thus, there is a negative relationship between GDP and price fluctuations in commodities derived from natural resources. Kalimullina and Orlov (31) and Dahl et al. (32) examined the relationship between decreased forest resources and efforts to improve income equality and alleviate poverty in different nations. The disparity in per capita GDP is a major factor in the degradation of natural resource sustainability. Numerous studies (33, 34) employing a wide range of statistical and economic methods have been carried out to gauge the impact. According to the findings, natural resource volatility is significantly influenced by efforts to reduce income and economic disparity. Trade gains are contingent on the long-term viability of economic globalization. This topic has been explored by researchers such as Gholizade et al. (35) and Menkeh (36), who looked at the balance of trade from the viewpoints of global income chains and GDP. Various statistical methods have warned us that as globalization advances, it will negatively impact the instability of natural resource availability. The findings show that increasing GDP brings economic advantages and considerably affects the volatility of prices for natural resources. Instead, Menkeh (36) provide evidence for a robust connection between GDP and the volatility of prices for natural resources. Both Tang et al. (37) and Su et al. (38) used the curse of reinterpretation to investigate the connection between human capital, GDP incentives, and natural resources. The elimination of income distortion is another benefit of the equitable distribution of natural resources. The empirical evidence suggests that the essential margins for natural resources are preserved through statistical and strategic approaches. The dynamic and cross-sectional panel estimates show that natural resource volatility is a major contributor to GDP. Business innovation and Africa's abundant natural resources were evaluated by Hai Ming et al. (39) using sustainable GDP's broad perspectives. Countries with abundant natural resources are seen as having weaker GDP incentives. As the mixed impact modeling approach enumerates, the damaging levels are also larger due to political and institutional abnormalities. Insights showed that a rise in GDP might mitigate natural resource volatility.

### Human capital and natural resources

Societal contribution is crucial in both sustainable and unsustainable ways for the longevity of natural resources. Human labor is crucial to creating such vast amounts of riches for the people of countries like China. Chinese researchers Li et al. (40) and Nasir et al. (41) looked into the potential of natural resources for holistic, sustainable development and societal benefit. By combining theoretical and strategic approaches, natural resources can be crafted as potent variables based on their social impact. Research demonstrates that social participation considerably impacts the price fluctuations of natural resources. Mngumi et al. (42) analyzed the connection between the value of natural resource price volatility and social contributions made by institutions and communities. Various tactical and theoretical evaluations have been placed on quantifying societal contributions' impacts on resource price volatility. According to the results, the value of natural resources and the viability of production are both boosted by the introduction and widespread adoption of important policies of social contribution. Du et al. (43) and Wei et al. (44) studied governance, information openness, and social media communication. Different tiers of social media efficiently reveal the robustness of social involvement. A variety of surveys and statistical methods are used to calculate the estimated value of a person's social contribution. According to the results, social participation has a significant and favorable effect on the price volatility of natural resources. Similar research has been conducted by Shao et al. (45) to investigate the environmental and societal factors that influence the development of South Africa's natural resource sectors. As such, the major goal of social investment is to ensure the long-term viability and export and worldwide market values of natural resource assets. Applying statistical and strategic methods, it has been claimed that social contribution can positively impact. The research showed that societal contribution was the most important factor when pricing natural resources. The tales of the connection between social contribution and the management of natural resource pricing were studied by Bilal et al. (46). As the economy moves toward sustainability, stabilizing natural resource prices and ensuring their continued supply require contributions from many different areas of society. The results showed that social participation significantly reduced the price volatility of natural resources.

The human capital index influences the volatility of prices for natural resources. Personal fortitude, emotional and physical wellbeing, life experiences, originality, problem-solving prowess, technical competence, and academic achievement are all components of human capital. Human capital development impacts how efficiently a company utilizes its resources. The demand for, and the volatility in the price of, natural resources are affected by human capital's effects on the economy. Zhang et al. (47) analyzed the correlation between technological progress, human capital, economic growth, and the volatility of the prices of natural resources. In attempting to evaluate the connection between these variables, statistical and econometric methods were used. It was found that there was a positive and statistically significant correlation between the human capital index and the natural resource commodity index. Public education investment, human capital accumulation, and the volatility of natural resource prices have all been studied by Chen et al. (48). These issues can be tackled by incorporating complementary components of natural resources and human capital with statistical methods. Investing in a country's human capital through health initiatives or new educational institutions has been shown to boost economic activity. For these tasks to be carried out, various resources, such as raw materials from natural sources or energy resources, are needed. Since people are using more of these things, the prices of these things have gone up. Thus, the volatility in the cost of natural resources is positively correlated with increases in human capital. These findings are at odds with those of (49), who found that a more productive workforce could replace technologies and make efficient use of natural resources thanks to investments in human capital. With less demand, natural resource prices fall; as a result, human capital drags the natural resources market's inherent instability. The interconnectedness of SSA's industrialization, human capital, and natural resource rents was investigated.

### Inflation and natural resource price volatility

It is commonly accepted that inflation is bad for the expansion of any economy and the cost of most goods and services. Because of the same issues with sustainability and maintainability, natural resource inflation is consistent across the board. Pan et al. (50) investigated inflation's impact on oil prices. Oil, gas, and other natural resource price differences result from inflation expectations and forecasts. Fundamental statistical and strategic methods have been used to estimate inflation's effects. The results demonstrated that natural resource prices increased as a result of inflation in the country. Therefore, commodity prices benefit from inflation. However, the interpretation of the relationship between inflation, natural resource prices, and international financial markets by Latif et al. (51) runs counter to these conclusions. Inflation fluctuates and has a detrimental effect on the world's natural resources, which has a dynamic impact on global financial markets. Inflation, natural resource pricing, ecological withdrawals, and FDI were all studied by Zhuo and Qamruzzaman (52). A number of econometric and statistical methods have been used to evaluate inflation's effects on the cost of natural resources. The results showed that inflation helps natural resources. The policies and management of natural resource monitoring programs were assessed by Dhal et al. (53). Several elements are considered while expanding inflation and management monitoring programs to account for price fluctuations in natural resources. The impact of macroeconomic conditions on the prices of natural resources has been examined using multiple regression analysis. Inflation was found to have a considerable and favorable effect on the prices of natural resources. Despite this, Nasir et al. (54) investigated the link between inflation's hedging properties, different types of international assets, and the volatility of the price of natural resources in South Africa. They discovered that inflation has a positive but negligible effect on the value of these commodities.

### Foreign direct investment and natural resources price volatility

Foreign direct investment's influence on the environment is contentious; there is evidence for both positive and bad outcomes. It has been recognized by The World Bank (55) that countries dependent on extractive sectors have seen significant increases in natural resource rents but that this growth is not sustainable unless these governments invest in productive assets. Xia et al. (56) found that one can look at the impact of natural resources from the supply or demand side. The first is the demand side and growth channel (determinants). Foreign direct investment (FDI) in the primary, manufacturing, and services sectors may have varied effects on economic development, a point addressed by Abbasi et al. (57). The Foreign Direct Investment (FDI) inflow to all sectors served as independent variables, and the Average Real Annual Per Capita Growth Rate served as the dependent variable in an analysis conducted by Mehmood (58) utilizing panel data for 47 nations across diverse regions. However Zubair et al. (59), pointed out that FDI in the manufacturing and service sectors promotes economic growth, while FDI in the primary industries negatively affects economic growth. A decrease in GDP growth of between −0.17 percentage points and −0.32 percentage points was found for every one percentage point rise in primary sector FDI. Using panel data analysis, Zhao et al. (60) investigated the factors that determined the GCC region's FDI location from 1980 to the present. According to findings, oil reserves, a proxy for natural resources, have a strikingly negative effect on foreign direct investment (61). However, natural resources were shown to be one of the most appealing factors of MNCs in studies of the factors determining the foreign direct investment a country receives. Hao et al. (62) used panel data analysis on 22 African nations between 1984 and 2000 to show that the ratio of mineral and oil exports to total exports is a significant proxy of natural resources in attracting FDI inflows. According to Zhang et al. (63) research, a rise of 0.65% in the FDI ratio follows a one-standard-deviation increase in natural resources. Similarly Zubair et al. (59), looked at the demand side characteristics that influenced FDI in 45 African nations between 1980 and 2007. Hailue discovered a favorable and statistically significant relationship between the FDI ratio and natural resources (mineral depletion as a percentage of GNI). The effect of a 1% increase in natural resources ranges from 0.50 to 0.19%. Natural resources, along with institutions, openness, and agglomeration, were found to be the most important factors in attracting FDI to this area by Dong et al. (64), who used unique panel data to investigate the determinants of FDI inflows to 25 transition economies between 1990 and 2018.

Since there is abundant empirical evidence linking GDP growth or per capita income increases to lower CO_2_ emissions in the reviewed literature, existing research has failed to give conclusive proof because they have produced conflicting results; some have discovered a positive correlation, while others have found a negative one. Consequently, reexamining this nexus is necessary for developing an all-encompassing economic policy in China. In addition, many reports investigate the connection between the price fluctuations of natural resource commodities and greenhouse gas releases. Despite the critical importance, no previous research has established a link between natural resource commodity price volatility and green recovery. Further, research and development's impact on economies and environmental quality has been extensively studied.

## Data and methods

The present study summarizes the core factors of natural resource price volatility for the top emerging economies such as China from 1995 to 2020. However, the data for selected variables have been taken from various sources, i.e., World Development Indicators (WDI) and GCSI for the selected period. Similarly, this study measures the green recovery *via* the level of carbon emissions in China and data from the GCSI. Furthermore, economic growth (GDP) data is collected from the WDI in current US dollars. Similarly, the human capital index data is being measured by gross enrolment in primary, secondary and tertiary education and taken from WDI. Foreign direct investment inflow data has been collected from the WDI and measured in % of growth. Data on inflation in consumer prices (annual %) also have been taken from the WDI. The data on natural resource price volatility has been collected from investing investing.com.

**Figure 1:**
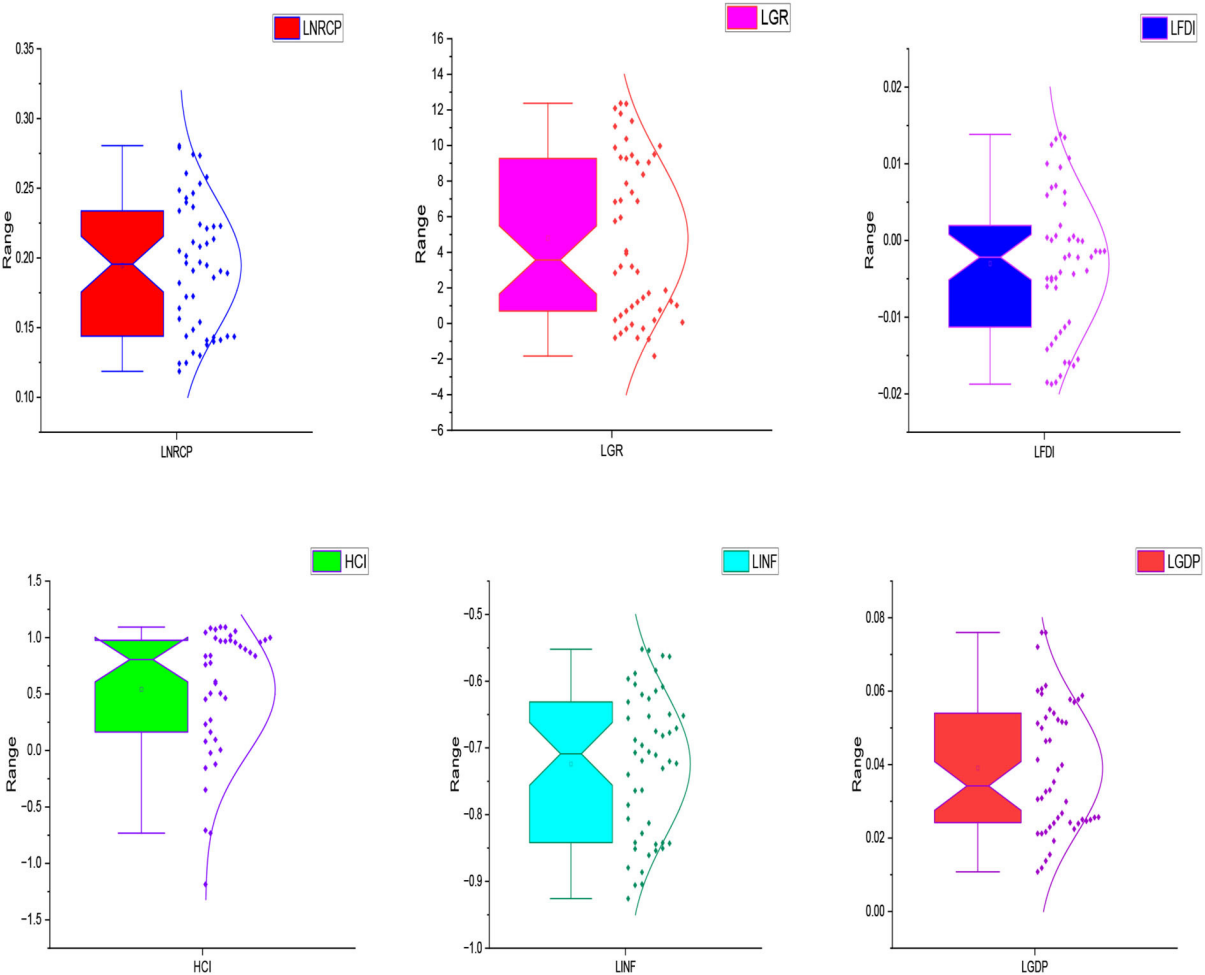
Box plots of the selected variables.

However, before moving forward to the estimation strategy and further empirical analysis, it is necessary to draw some graphs to check out the data response. Therefore, Figure 1 consists of Box Plots of the selected variables.

However, on behalf of outcomes, the current study tries to propose a decent model consisting of green recovery, economic growth (GDPG), inflation, foreign direct investment, inflation, and the human capital index as the determinants of natural resource price volatility. Similarly, the function can be written as well,


(1) $$\text{NRCP}=\text{f}({\text{β}}_{0},{\text{GR}}^{\text{β}1},{\text{HCI}}^{\text{β}2},{\text{INF}}^{\text{β}3},{\text{GDPG}}^{\text{β}4},{\text{FDI}}^{\text{β}5},{\text{e}}^{\text{μ}})$$

By taking a natural log, we can transform a given function into a linear equation, and it can be written as well,


(2) $$\begin{array}{l} {\text{LNRCP}}_{\text{t}}={\text{β}}_{\text{0}}+{\text{β}}_{\text{1}}{\text{LGR}}_{\text{t}}+{\text{β}}_{\text{2}}{\text{HCI}}_{\text{t}}+{\text{β}}_{\text{3}}{\text{LINF}}_{\text{t}}\\ \;+{\text{β}}_{\text{4}}{\text{LGDPG}}_{\text{t}}+{\text{β}}_{\text{5}}{\text{LFDI}}_{\text{t}}+{\text{μ}}_{\text{t}} \end{array}$$

In Eq. (2), LNRCP, LGR, LINF, LGDPG, and LFDI refer to the natural log of natural resource price volatility, green recovery, inflation, economic growth (GDP) and foreign direct investment. Whereas t refers to the time period, and μ is the error term.

### Estimation strategy

#### Unit root tests

In this investigation, we employ a combination of the Zivot-Andrews test with one structural break and the Augmented Dickey-Fuller (ADF) test of Dickey and Fuller (65). The ADF test is used to verify the degree to which the datasets have been integrated. One of the major flaws of this method is that it may misinterpret procedural breaches in a set of facts with a non-stationary origin. Conversely, the null hypothesis of unit origin cannot be supported if the sequence under study has a structural divide. To remedy this, we employ the Zivot - Andrews root test, which requires only a single structural break.

#### Bayer and hanck co-integration test

Numerous methods of testing for a cointegration relationship between variables have been described in the econometric literature. Previous literature, such as Johansen (66), and Katircioglu (67), suggests that a linear stationary arrangement among a number of series indicates a long-run relationship between them. In addition, the results of the many tests for cointegration and non-cointegration were condensed into fewer, more manageable null hypotheses. Similarly, determining the individual test statistics (68) might lead to more convincing results. The following general mathematical form was utilized in this analysis, which followed the Bayer and Hank co-integration approach (Eq. 3 and 4),


(3) G −JOH =−2[log(Prob.EG)+ (Prob.JOH)]

(4) $$\begin{array}{l} \text{EG}-\text{JOH}-\text{BO}-\text{BDM = }-2[\text{log}(\text{Prob}.\text{EG})\\ +(\text{Prob}.\text{JOH})+(\text{Prob}.\text{BO})+(\text{Prob}.\text{BDM})] \end{array}$$

Likewise, P_rob.EG_, P_rob.JOH_, P_rob.BO_, and P_rob.BDM_ is symbolized by individual probabilities of each test.

However, before moving forward to a long-term estimation strategy, it is necessary to elaborate here why this study focuses on the ARDL test instead of OLS or FMOLS estimators. It is a general selection criterion that if all variables are integrated at the level, then the study must select the OLS technique for empirical estimation. Similarly, if all variables are integrated at the first difference, it should be compulsory to adopt the FMOLS estimator. Finally, when all variables are the mixture of level and first difference, OLS & FMOLS may provide biased and inconsistent estimates. Therefore, to escape from unbiased and inconsistent estimates, this study prefers to ARDL estimator.

#### ARDL estimation

To evaluate the stability of the connection between variables over time, we combine the bounds test with the dynamic simulated ARDL method. We used the ARDL bound test for cointegration to examine the short- and long-term association between the variables in this analysis. The equation we used as a model is as follows.


(5) $$\begin{array}{l} \text{Δ}{\text{NRCP}}_{\text{t}}={\text{β}}_{\text{0}}\text{+}\sum_{\text{i=0}}^{\upsilon }\beta_{\text{1}}\text{Δ}{\left(\text{GR}\right)}_{\text{t}}\text{+}\sum_{\text{i=1}}^{\text{t}}\beta_{\text{2}}\text{Δ}{\left(\text{HCI}\right)}_{\text{t}-\text{1}}\\ \text{ +}\sum_{\text{i=0}}^{\upsilon }\beta_{\text{3}}\text{ Δ}{\left(\text{GDP}\right)}_{\text{t}-\text{1}}\text{+}\sum_{\text{i=0}}^{\text{j}}\beta_{\text{4}}\text{Δ}{\left(\text{FDI}\right)}_{\text{t}-\text{1}}\\ \text{ +}\sum_{\text{i=0}}^{\text{j}}\beta_{\text{5}}\text{Δ}{\left(\text{INF}\right)}_{\text{t}-\text{1}}\text{+}{\text{β}}_{\text{1}}\;{\left(\text{GR}\right)}_{\text{t}-\text{1}}\text{+}{\text{β}}_{\text{2}}{\left(\text{HCI}\right)}_{\text{t}-\text{1}}\\ \text{ +}{\text{β}}_{\text{3}}{\left(\text{GDP}\right)}_{\text{t}-\text{1}}\text{+}{\text{β}}_{\text{4}}{\left(\text{FDI}\right)}_{\text{t}-\text{1}}\text{+}{\text{β}}_{\text{5}}{\left(\text{INF}\right)}_{\text{t}-\text{1}}\text{+}\varepsilon_{\text{t}} \end{array}$$

Where denotes the initial term in the differential: Natural resources price volatility (NRCP), green recovery (GR), human capital index (HCI), gross domestic product (GDP) growth (GDP), foreign direct investment (FDI) growth (INF), and inflation (INF) disclose all of the monetary variables. As determined by Zucchini (69)'s information criteria, the optimal lag decisions are denoted by t1. This paper analyzes the above equation to determine the short-term and long-term relations between the variables. The possible outcomes and null hypotheses for the ARDL bound test are as follows:


(6) $$\begin{array}{l} {\text{H}}_{0}={\text{ϕ}}_{1}={\text{ϕ}}_{2}={\text{ϕ}}_{3}={\text{ϕ}}_{4}={\text{ϕ}}_{5}={\text{ϕ}}_{6}=0 \end{array}$$

(7) $$\begin{array}{l} {\text{H}}_{1}={\text{ϕ}}_{1}={\text{ϕ}}_{2}={\text{ϕ}}_{3}={\text{ϕ}}_{4}={\text{ϕ}}_{5}={\text{ϕ}}_{6}=0 \end{array}$$

#### Error correction-based granger causality analysis

Since the study's two econometric methods (MS-ECM and OLS) cannot trace the causality relationship between the variables, we resort to Granger causality. In the absence of evidence of co-integration of the variables, the vector autoregression test in the first differences is defined as the basis for the Granger causality test. However, a one-period lag error correction factor must be included in the granger causal test mode if we find evidence of co-integration (ECT-1). If the results confirm the existence of a long-term correlation between the designated environmental quality indicators, we can proceed to quantify the VECM given in Eqs. 15–20 by building upon the work of Engle and Granger (70).


(8) $$\begin{array}{l} \text{Δ}{\text{NRCP}}_{\text{t}}=\delta_{\text{0}}\text{+}\sum_{\text{k}=1}^{\text{n1}}\delta_{\text{1}}\text{kΔ}{\text{GR}}_{\text{t}-\text{k}}\\ \text{+}\sum_{\text{k}=1}^{\text{n2}}\delta_{\text{2}}\text{kΔ}{\text{HCI}}_{\text{t}-\text{k}}\text{+}\sum_{\text{k}=1}^{\text{n3}}\delta_{\text{3}}\;\text{kΔ}{\text{GDP}}_{\text{t}-\text{k}}\\ \text{+}\sum_{\text{k}=1}^{\text{n4}}\delta_{\text{4}}\text{kΔ}{\text{FDI}}_{\text{t}-\text{k}}\text{+ +}\sum_{\text{k}=1}^{\text{n5}}\delta_{\text{5}}\text{kΔ}{\text{INF}}_{\text{t}-\text{k}}\\ \text{+}\tau {\text{ECT}}_{\text{t}-\text{1}}\text{+}{\text{μ}}_{\text{t}} \end{array}$$

(9) $$\begin{array}{l} \text{Δ}{\text{GR}}_{\text{t}}=\delta_{\text{0}}\text{+}\sum_{\text{k}=1}^{\text{n1}}\delta_{\text{1}}\text{kΔ}{\text{GR}}_{\text{t}-\text{k}}\\ \text{+}\sum_{\text{k}=1}^{\text{n2}}\delta_{\text{2}}\text{kΔ}{\text{HCI}}_{\text{t}-\text{k}}\text{+}\sum_{\text{k}=1}^{\text{n3}}\delta_{\text{3}}\;\text{kΔ}{\text{GDP}}_{\text{t}-\text{k}}\\ \text{+}\sum_{\text{k}=1}^{\text{n4}}\delta_{\text{4}}\text{kΔ}{\text{FDI}}_{\text{t}-\text{k}}\text{+ +}\sum_{\text{k}=1}^{\text{n5}}\delta_{\text{5}}\text{kΔ}{\text{INF}}_{\text{t}-\text{k}}\\ \text{+}\tau {\text{ECT}}_{\text{t}-\text{1}}\text{+}{\text{μ}}_{\text{t}} \end{array}$$

(10) $$\begin{array}{l} \text{Δ}{\text{HCI}}_{\text{t}}=\delta_{\text{0}}\text{+}\sum_{\text{k}=1}^{\text{n1}}\delta_{\text{1}}\text{kΔ}{\text{GR}}_{\text{t}-\text{k}}\\ \text{+}\sum_{\text{k}=1}^{\text{n2}}\delta_{\text{2}}\text{kΔ}{\text{HCI}}_{\text{t}-\text{k}}\text{+}\sum_{\text{k}=1}^{\text{n3}}\delta_{\text{3}}\;\text{kΔ}{\text{GDP}}_{\text{t}-\text{k}}\\ \text{+}\sum_{\text{k}=1}^{\text{n4}}\delta_{\text{4}}\text{kΔ}{\text{FDI}}_{\text{t}-\text{k}}\text{+ +}\sum_{\text{k}=1}^{\text{n5}}\delta_{\text{5}}\text{kΔ}{\text{INF}}_{\text{t}-\text{k}}\\ \text{+}\tau {\text{ECT}}_{\text{t}-\text{1}}\text{+}{\text{μ}}_{\text{t}} \end{array}$$

(11) $$\begin{array}{l} \text{Δ}{\text{GDP}}_{\text{t}}=\delta_{\text{0}}\text{+}\sum_{\text{k}=1}^{\text{n1}}\delta_{\text{1}}\text{kΔ}{\text{GR}}_{\text{t}-\text{k}}\\ \text{+}\sum_{\text{k}=1}^{\text{n2}}\delta_{\text{2}}\text{kΔ}{\text{HCI}}_{\text{t}-\text{k}}\text{+}\sum_{\text{k}=1}^{\text{n3}}\delta_{\text{3}}\;\text{kΔ}{\text{GDP}}_{\text{t}-\text{k}}\\ \text{+}\sum_{\text{k}=1}^{\text{n4}}\delta_{\text{4}}\text{kΔ}{\text{FDI}}_{\text{t}-\text{k}}\text{+ +}\sum_{\text{k}=1}^{\text{n5}}\delta_{\text{5}}\text{kΔ}{\text{INF}}_{\text{t}-\text{k}}\\ \text{+}\tau {\text{ECT}}_{\text{t}-\text{1}}\text{+}{\text{μ}}_{\text{t}} \end{array}$$

(12) $$\begin{array}{l} \text{Δ}{\text{FDI}}_{\text{t}}=\delta_{\text{0}}\text{+}\sum_{\text{k}=1}^{\text{n1}}\delta_{\text{1}}\text{kΔ}{\text{GR}}_{\text{t}-\text{k}}\\ \text{+}\sum_{\text{k}=1}^{\text{n2}}\delta_{\text{2}}\text{kΔ}{\text{HCI}}_{\text{t}-\text{k}}\text{+}\sum_{\text{k}=1}^{\text{n3}}\delta_{\text{3}}\;\text{kΔ}{\text{GDP}}_{\text{t}-\text{k}}\\ \text{+}\sum_{\text{k}=1}^{\text{n4}}\delta_{\text{4}}\text{kΔ}{\text{FDI}}_{\text{t}-\text{k}}\text{+ +}\sum_{\text{k}=1}^{\text{n5}}\delta_{\text{5}}\text{kΔ}{\text{INF}}_{\text{t}-\text{k}}\\ \text{+}\tau {\text{ECT}}_{\text{t}-\text{1}}\text{+}{\text{μ}}_{\text{t}} \end{array}$$

(13) $$\begin{array}{l} \text{Δ}{\text{INF}}_{\text{t}}=\delta_{\text{0}}\text{+}\sum_{\text{k}=1}^{\text{n1}}\delta_{\text{1}}\text{kΔ}{\text{GR}}_{\text{t}-\text{k}}\\ \text{+}\sum_{\text{k}=1}^{\text{n2}}\delta_{\text{2}}\text{kΔ}{\text{HCI}}_{\text{t}-\text{k}}\text{+}\sum_{\text{k}=1}^{\text{n3}}\delta_{\text{3}}\;\text{kΔ}{\text{GDP}}_{\text{t}-\text{k}}\\ \text{+}\sum_{\text{k}=1}^{\text{n4}}\delta_{\text{4}}\text{kΔ}{\text{FDI}}_{\text{t}-\text{k}}\text{+ +}\sum_{\text{k}=1}^{\text{n5}}\delta_{\text{5}}\text{kΔ}{\text{INF}}_{\text{t}-\text{k}}\\ \text{+}\tau {\text{ECT}}_{\text{t}-\text{1}}\text{+}{\text{μ}}_{\text{t}} \end{array}$$

Here, we use the lagged error correction tool ECT-1, which is based on long-run Granger causality, τ used to find equilibrium after a shock (S) to the system.

## Results and discussion

The authors have checked the descriptive statistics, including the average, standard deviation, range, and extremes. Furthermore, the total number of observations is given. The data provided determined that the median NRCP was 80.873 percent, and the median GR was 4.031 percent. Also revealed by the numbers is that while GDPG was 2.119 percent, the mean HCI was only 0.289%. Lastly, the data showed that the average FDI was 4.337 percent (Table 1).

**Table 1:** Descriptive statistics.

**Variable**	**Obs**	**Mean**	**Std. Dev**.	**Min**	**Max**
LNRCP	52	80.873	3.351	72.468	85.145
LGR	52	4.308	1.390	2.196	7.446
HCI	52	0.289	1.075	0.124	0.501
LINF	52	−3.728	−0.337	−1.946	−6.442
LGDPG	52	2.119	0.466	1.157	6.333
LFDI	52	4.337	0.607	2.454	9.538

The writers have also used the correlation matrix to examine the interconnections between different concepts. The results showed a positive relationship between NRCP and all of the predictors. The results are displayed in Table 2.

**Table 2:** Matrix of correlations.

**Variables**	**NRCP**	**EXP**	**HCI**	**GDPG**	**FDI**	**INF**
LNRCP	1					
LGR	0.32315	1				
HCI	0.6771	0.38745	1			
LGDPG	0.4016	0.29925	0.3738	1		
LFDI	0.4479	0.03635	0.4211	0.2415	1	
LINF	0.31955	0.01115	0.2688	0.1386	0.2667	1

The variance VIF was also used to examine the multicollinearity among the variables in this investigation. Results showed that VIF values are <5, indicating no multicollinearity. We can see the VIF outcomes in Table 3.

**Table 3:** Variance inflation factor.

**Variables**	**VIF**	**1/VIF**
LGR	4.218	0.261
HCI	2.119	0.519
LGDPG	1.146	0.961
LFDI	3.066	0.359
LINF	3.056	0.452
Mean VIF	2.512	.

Given that the study's proper model has already been determined by the stationarity of the variable in question, the ADF test is also conducted to double-check this assumption. According to the data, NRCP, GR, and HCI are level, whereas GDPG, INF and FDI are static at the first difference. Because of this, the ARDL model is valid. You may see the ADF outcomes in Table 4. However, Table 5 shows the structural break unit root test by Zivote.

**Table 4:** Unit root test.

**Variable**		**ADF test**		**PP test**	
		**t-statistics**	***p*-values**	**t-statistics**	***p*-values**
LNRCP	I (1)	−4.218	0.001	−9.333	0.000
LGR	I (1)	−5.240	0.000	−4.542	0.005
HCI	I (1)	−4.105	0.009	−7.279	0.001
LINF	I (0)	−2.342	0.000	−5.449	0.002
LGDPG	I (0)	−4.629	0.000	−11.963	0.000
LFDI	I (0)	−5.346	0.000	−3.198	0.004

**Table 5:** Unit root test.

	**ZA Test**			
**Variable**	**Level**		**1st difference**	
	**Intercept**	**Structural break**	**Intercept**	**Structural break**
LNRCP	−1.669**	33	−1.952**	31
LGI	−3.348*	31	−5.833*	28
HCI	−5.963*	12	−4.199*	9
LINF	−3.902*	28	−5.963*	24
LGDP	−5.473*	21	−6.117*	17
LFDI	−2.678**	33	−1.456**	25

* and ** show the significance level at 1 and 5%, respectively.

As shown by the unit root test results (Table 5), the further analysis does not necessitate using any variables with an order of integration higher than 1. Each variable in all three nations is either level or first difference stationary.

This study uses the Akaik Lag structure criteria, and results are given in Appendix A. Table 6 shows the ARDL limits test of cointegration for the selected model. The table displays the 5% significance level and the F-statistic value. Pesaran and Shin (71) and Dinda and Coondoo (72) are the sources used to get the reported critical values. We assume an I(0) ARDL model for the I(0) critical values and an I(1) model for the I(1) values (1).

**Table 6:** ARDL bound test.

**Model**	**F-stat**	**Lag**	**Level of significance**	**Bound test critical values**	
				**I (0)**	**I (1)**
NRCP/(GR,HCI,GDPG, INF,FDI)	5.77	4	1%	6.65	7.34
			5%	5.37	5.71
			10%	4.76	5.23

The conditional volatility of natural resource rents is the independent variable in the equation used to determine F (GDP/Rents), the F-statistics value. However, this study also tries to investigate the cointegration by Jhonson and Bayer & hank cointegration. The results are given in Tables 7, 8.

**Table 7:** Johnson co-integration results.

**Trace Statistics**				
**Hypothesized** **No. of CE (s)**	**Eigenvalue**	**Trace** **Statistic**	**0.05** **Critical Value**	Prob.**
None*	0.756	315.647	163.477	0.000
At most, 1*	0.799	209.599	125.632	0.000
At most, 2*	0.773	145.332	105.441	0.000
At most, 3*	0.761	95.638	70.683	0.000
At most 4*	0.690	59.742	35.119	0.000
At most 5*	0.520	25.221	13.852	0.000
At most 6*	0.336	8.857	5.669	0.003
**Hypothesized** **No. of CE (s)**	**Eigenvalue**	**Max-eigen** **statistic**	**0.05** **critical value**	**Prob**.**
None*	0.923	90.633	47.312	0.000
At most 1*	0.890	71.111	38.456	0.000
At most 2*	0.799	59.205	31.324	0.000
At most 3*	0.749	49.321	25.485	0.000
At most 4*	0.601	25.333	19.136	0.007
At most 5*	0.563	17.359	11.652	0.008
At most 6*	0.399	8.263	4.402	0.003

* and ** symbols indicate 1% and 5% significance level.

**Table 8:** Bayer and hank cointegration.

**Quantified specifications**	**EG-JOH**	**EG-JOH-BOBDM**	**Co-integration**
	9.8132*	16.8065**	Exists

* and ** shows the level of significance at 1 and 5%, respectively.

### Short-run and long run coefficients

Based on the ARDL model's findings, exports, GDP growth, human capital, and FDI are all positively correlated with natural resource price volatility in China over the short term. The data determined that for every one-unit increase in GR, the NRCP would rise by 1.920 units, and for every one-unit increase in HCI, the NRCP would rise by 0.820 units. Statistics also reveal that for every one-unit increase in GDPG, the NRCP will increase by 4.291 units and vice versa, while for every one-unit increase in FDI, the NRCP will increase by 1.108 units and vice versa. The short-term correlation among the constructs is displayed in Table 9. However, it is necessary to discuss the ECM value here, which many readers can think about. ECM value refers to the speed of adjustment from the short to the long run of selected variables. In other words, it explains how much time it will require to converge from the short to the long run. According to the given value, it will take less than half a year to converge into the long run.

**Table 9:** Short run coefficients.

**Variable**	**Coefficient**	**Std. Error**	**t-Statistic**
ΔLGR	2.016^*^	0.861	2.45805
ΔHCI	0.861^*^	0.21105	4.28295
ΔLGDPG	4.505^*^	1.6716	2.8287
ΔLFDI	1.163^*^	0.21945	5.56395
ΔLINF	2.178^*^	1.2327	1.85535
CointEq (−1)^*^	0.340^*^	0.11235	3.1794
R-squared	0.505031	Mean dependent var	
Adjusted R-squared	0.492630	S.D. dependent var	

* for 1% significance separately.

Long-term ARDL model results show a positive relationship between exports, human capital, GDP growth, and FDI in China, and thus natural resources price volatility. The association between the variables over time is displayed in Table 10. Similarly, green recovery is found to have a positive and statistically significant relationship with natural resource price volatility (LNRCP). An average of 2.490% rise in the long run and 1.920% in the short run is caused in LNRCP with a 1% increase in green recovery. These results suggest that the increase in green recovery is the cause of extracting natural resources. Due to the overexploitation of natural resources, China's bio-capacity may be negatively impacted, which may explain the positive correlation between resource price volatility and green recovery in the country. In addition, the increase in GR is the root cause of natural resource price swings (73, 74). Due largely to the construction of new infrastructure, China has been among the newly industrialized countries since 2000, with its share of the industrialized economy devoted to material extraction on the rise. Rapid growth in the Chinese economy is largely attributable to the country's expanding industrialization, yet this development has been linked to the unsustainable depletion of natural resources and reliance on foreign fossil fuels (75, 76).

**Table 10:** Long-term coefficients.

**Variable**	**Coefficient**	**Std. error**	**t-statistic**
LGR	2.490^*^	0.641	3.884
HCI	3.369^*^	1.262	2.669
LINF	4.248^*^	1.883	2.255
LGDPG	1.584^*^	0.535	2.960
LFDI	3.561^*^	0.999	3.564
C	0.275**	0.097	2.835

* symbol indicates 1% significance separately.

The coefficient of the human capital index demonstrates a similar positive relationship between that indicator and the price volatility of natural resources. According to the formula, a long-term increase of 3.369% in LNRCP can be expected from a one percentage point gain in this factor. Human capital is found to have a substantial beneficial effect on NRCP. If we boost investment in people by only 1%, productivity will rise by 3.369%. The benefits of investing in people don't change depending on the details. The findings support the argument made by Nassani et al. (77), who noted that abundant natural resources and human capital has a marginal influence.

Additionally, the study found that inflation in the country influences the volatility of pricing for natural resources in a beneficial way. Since the price of natural resources rises in response to an uptick in business activity and production, inflation is a common result. Producers are encouraged by the rising pricing of natural resources to expand output in response to current and anticipated economic needs. Our findings are consistent with those of Yang et al. (73), who found that when inflation is present, there is an increase in the amount of money in circulation. Because natural resources are limited in number or quantity, their prices rise rather than stabilize. These findings are consistent with theirs and consistent with Cevik et al. (78), these findings are likewise consistent with theirs. The research looked at how inflation affected the pricing of natural resources and concluded that governments tend to undertake building or development projects during inflation. All these buildings and improving endeavors need a substantial quantity of natural resources and products derived from natural resources (79). The prices of relevant natural resources rise in tandem with their demand. Therefore, a high inflation rate drives up the cost of essential materials.

Similarly, economic growth is another factor that can manage the price volatility of natural resources. It is positively associated with natural resources price volatility, which infers that a 1% rise in this factor would cause to increase in NRCP by 1.584% in the long run. These findings corroborate the favorable correlation between NRCP and GDP expansion observed in prior empirical work by Gao et al. (80). By providing the energy and other resources necessary for industrial production, the economy's expansion boosts NRCP and, in turn, the region's economic growth. According to previous research, natural gas has been shown to impact economic growth positively. Several recent studies, including those by Yang et al. (73), Shahbaz et al., (81), and Umar et al. (74), have found that natural gas has a positive effect on economic development in a number of countries.

Last but not the least, foreign direct investment is also positively associated with natural resource price volatility. The investment variable had a significant positive relationship with the price volatility of natural resources. A 1% increase in FDI causes the price volatility of natural resources to increase by 3.561% in the long run. Finance institutions have inherent reasons to handle resources well, and the agreement between individual and group rationality makes accessible investment often efficiently addressed. Reason number two: everywhere you look, people have competing interests between preventing climate change and keeping the money they get from exploiting local resources. This result makes sense and is consistent with Ahmed and Sarkodie (82) case study of GCC economies.

#### Stability tests

It is necessary to investigate the stability of the selected model; therefore, the current study uses the χ2 test, ARCH test, WHITE test and REMSAY test. All the given tests show reliable outcomes, and there was no evidence for the serial correlation in the selected model (Table 11).

**Table 11:** Diagnostic tests.

**χ2SERIAL**	**χ2ARCH**	**χ2WHITE**	**χ2REMSAY**
F-statistics	F-statistics	F-statistics	F-statistics
6.5632 [0.4123]	0.3645 [0.1114]	1.33254 [0.3819]	0.6349 [0.3298]

### Granger causality testing

Table 12 demonstrates how the VECM Granger is used to find the causation interaction between the studied variables and to dissect the trajectory of the interaction over the short and long terms. There exists a bi-directional relationship between economic growth and natural resource price volatility. It infers that any significant change in economic growth would cause of change in NRCP. Such results also validate the long-run results by estimators. Besides, there exists a bi-directional association between green recovery and GDP growth. However, there exists the uni-directional causality from GI to foreign direct investment. Furthermore, the uni-directional causality between FDI and green recovery and economic growth was found. Also, a uni-directional association exists between GDP to NRCP and green recovery.

**Table 12:** Short-run diagnostic tests.

**DV**	**Type of granger causality**						
	**Short run (lag)**						**Long run**
	**ΔLNRCP**	**ΔLGI**	**ΔHCI**	**ΔLINF**	**ΔLGDP**	**ΔLFDI**	**ECT-1**
	**F-statistics [** ***P*** **-values]**						**t-stat**
ΔLNRCP	-	1.36951 [0.3352]	1.70314 [0.1743]	1.17849 [0.1596]	12.3645* [0.0009]	1.145401 [0.3327]	−0.78963 [−3.3228]
ΔLGI	1.19464 [0.4900]	-	2.46340 [0.2441]	2.40326 [0.5613]	3.27093 [0.0310]	4.97596 [0.0412]	−0.56932 [−5.2612]
ΔHCI	1.3217 [0.4321]	0.81390 [0.6440]	–	1.39312 [0.3329]	21.4856* [0.0000]	0.39306 [0.3052]	−0.70536 [−1.3815]
ΔLINF	0.3541 [0.3658]	5.32780* [0.0002]	5.52403*** [0.0224]	-	0.67692 [0.1193]	0.18340 [0.2759]	0.928186 [0.6175]
ΔLGDP	6.9438 [0.0001]	8.9732 [0.0000]	11.374 [0.0000]	1.21765 [0.2222]	-	2.35832 [0.1263]	−0.34283 [−3.5021]
ΔLFDI	1.6914 [0.2963]	5.37542* [0.0021]	1.08952 [0.3073]	2.54276 [0.3611]	11.5069* [0.0000]	–	−0.97260 [−1.3742]

* and *** symbols indicate 1 and 10% significance separately.

## Conclusion and policy implications

The key focus of this study is the investigation of determinants of natural resource price volatility in China for the period 1971–2020 with annual frequency. Therefore, this study investigates the impact of green recovery, inflation, foreign direct investment, human capital index and inflation on NRCP in China. In order to estimate the objectives of the study, the current study employs the Autoregression Distributive Lag Model (ARDL). The obtained results show the selected variable's positive contribution toward the natural resources price volatility. On behalf of outcomes, this study suggests some imperative policies for the NRCP.

### Policy recommendations

Evidence from this study supports recommendations for policymakers, academics, and governors to consider in light of the potential link between economic success and natural resource price volatility. As a first step, the Chinese economy should ensure that aggregate demand and supply for natural resources are kept in check and that these resources are used effectively. For the sake of promoting economic growth and performance while mitigating the effects of natural resources price volatility, price regulation may be essential. Second, hedging of natural resources like petroleum reserves should be kept by a chosen few economies in order to eliminate price fluctuation. The result is less oil price volatility over a shorter time frame, which may be extended further into the future. More than that, it's important to pay close attention to economic activities because they affect the demand for and supply of natural resources and, thus, the prices at which they trade. In light of the aforementioned, hedging natural resources could mitigate the negative effects of natural resource volatility on the economy. Third, a policy of capping or freezing prices may be useful for stabilizing the economy and preventing the wild swings in output that can threaten long-term growth.

Second, changes to China's policy on natural resource commodity pricing would significantly impact the country's economic performance. This suggests that any policy shifts should consider the economy's current state. If policymakers and economists in a country are serious about ensuring that their efforts to boost the economy don't come at the expense of future generations, then they need to follow the recommendations presented in this paper. It instructs state and economic policymakers on how to keep natural resource prices stable through sound policies, plans, or strategies relating to both economic and non-economic matters, thereby improving the availability and quality of those resources for the benefit of present and future generations. The research argues that in order to stabilize natural resource pricing and ensure their continued production and conservation for long-term economic growth, policymakers and economists should prioritize rising prices, GDP, social contributions, and human capital.

### Future limitations

This study has a number of flaws that cast doubt on its overall reliability and validity. These caveats need to be addressed in follow-up research. This research looked at the correlation between the volatility of the prices of natural resources and a handful of limited economic parameters like inflation, GDP growth, and the human capital index. This reduces the study's rigor, thus future researchers should add more variables (such as social and political factors) to their models of natural resource price volatility. Second, all the information we have on how natural resource prices are affected by macroeconomic variables like inflation and GDP growth comes from only one developing country—China. Unique economic and social conditions prevail in China. Therefore, it's possible that the results of the Chinese study don't generalize. It is recommended that the analysis be performed in several different economies.

## Data availability statement

Publicly available datasets were analyzed in this study. This data can be found here: https://databank.worldbank.org/source/world-development-indicators.

## Author contributions

The author confirms being the sole contributor of this work and has approved it for publication.

## Appendix

**Appendix A:** Lag structure criteria.

	**AIC**	**SC**	**HQ**
0	–19.81583	–19.75519	–19.30973
1	–38.19509	–36.20892	–37.86657
2	–51.96479	–44.25036	–51.83240
3	–57.55020^*^	–47.90978^*^	–55.61519^*^

* Shows the level of significance at 1%.
